# Mitochondrial Dysfunction and Adipogenic Reduction by Prohibitin Silencing in 3T3-L1 Cells

**DOI:** 10.1371/journal.pone.0034315

**Published:** 2012-03-30

**Authors:** Dong Liu, Yiming Lin, Ting Kang, Bo Huang, Wei Xu, Minerva Garcia-Barrio, Moshood Olatinwo, Roland Matthews, Y. Eugene Chen, Winston E. Thompson

**Affiliations:** 1 Cardiovascular Research Institute, Morehouse School of Medicine, Atlanta, Georgia, United States of America; 2 Department of Physiology, Morehouse School of Medicine, Atlanta, Georgia, United States of America; 3 Department of Endocrinology, School of Medicine, Emory University, Atlanta, Georgia, United States of America; 4 Division of Cardiology, the First Affiliated Hospital, Nanchang University, Nanchang, China; 5 Department of Clinical Laboratory Sciences, the Second Affiliated Hospital, Nanchang University, Nanchang, China; 6 Department of Obstetrics and Gynecology, Morehouse School of Medicine, Atlanta, Georgia, United States of America; 7 Cardiovascular Center, University of Michigan, Ann Arbor, Michigan, United States of America; Medical College of Georgia, United States of America

## Abstract

Increase in mitochondrial biogenesis has been shown to accompany brown and white adipose cell differentiation. Prohibitins (PHBs), comprised of two evolutionarily conserved proteins, prohibitin-1 (PHB1) and prohibitin-2 (PHB2), are present in a high molecular-weight complex in the inner membrane of mitochondria. However, little is known about the effect of mitochondrial PHBs in adipogenesis. In the present study, we demonstrate that the levels of both PHB1 and PHB2 are significantly increased during adipogenesis of 3T3-L1 preadipocytes, especially in mitochondria. Knockdown of PHB1 or PHB2 by oligonucleotide siRNA significantly reduced the expression of adipogenic markers, the accumulation of lipids and the phosphorylation of extracellular signal-regulated kinases. In addition, fragmentation of mitochondrial reticulum, loss of mitochondrial cristae, reduction of mitochondrial content, impairment of mitochondrial complex I activity and excessive production of ROS were observed upon PHB-silencing in 3T3-L1 cells. Our results suggest that PHBs are critical mediators in promoting 3T3-L1 adipocyte differentiation and may be the potential targets for obesity therapies.

## Introduction

During the development of obesity, adipocytes become hypertrophic until a crucial cell size is reached, after which they further expand the fat mass by adipocyte hyperplasia [1], [2]. Preadipocytes, even from elderly humans, retain the capacity to differentiate *in vitro* [3]. Mitochondrial biogenesis, found during mouse 3T3-L1 preadipocyte differentiation, is accompanied by the remodeling of the mitochondrion and is considered to be a necessary adjustment because the cells become increasingly active in metabolism [4]. Enhancement of mitochondrial biogenesis during adipogenesis may be the result of activation or enhanced expression of nuclear encoded mitochondrial genes that are under the control of adipogenic transcription factors [5]. The essential role of mitochondrial biogenesis during adipogenesis is further confirmed by the observation that induction of mitochondrial dysfunction inhibits adipogenesis in 3T3-L1 preadipocytes [6], [7].

Mitochondria are extremely dynamic structures that fuse and divide continuously to adjust the shape and distribution of the mitochondrial network depending on cell type and energy demands, therefore playing critical roles in cell physiology. Prohibitin (PHB) proteins are highly expressed in cells that rely heavily on mitochondrial function [8]. PHBs comprise two evolutionarily conserved proteins, prohibitin-1 (PHB1) and prohibitin-2 (PHB2, a.k.a. REA). Both proteins associate in heterodimers in a high molecular-weight complex (∼1.2 MDa) in the inner membrane of mitochondria [9], [10], [11], [12], [13]. About 12 to 16 PHB heterodimers associate to form a ring-like structure at the mitochondrial inner membrane [11]. PHB1 and PHB2 are physically interactive [9], [14], [15] and functionally interdependent in various organisms [12], [14], [16], [17], [18]. The absence of either PHB does not affect the expression of the other, but results in its posttranslational degradation. Our previous work revealed that PHB1 is essential for stabilizing the mitochondrial integrity and membrane potential in human ovarian cancer cells and rat ovarian granulosa cells [19], [20]. Loss of PHBs brings about altered organization and reduced copy number of mtDNA, and unstabilized mitochondrial-encoded subunits of the respiratory chain [21]. Affected mtDNA within fragmented mitochondria may cause the disruption of OXPHOS and therefore promote the production of free radicals [22]. Indeed, lack of PHB1 results in increased levels of reactive oxygen species (ROS) in endothelial cells [23]. An increase in mitochondrial ROS generation is demonstrated to prevent preadipocyte differentiation through upregulation of C/EBPζ, an adipogenic repressor [24].

Increased intracellular expression and decreased extracellular secretion of PHBs have been observed during adipogenesis [25], [26]. A recent publication has shown that PHB deficiency in nematode markedly reduces mitochondrial membrane potential and fat content early in adulthood [27]. However, the effects of PHBs during adipogenesis in mammals are still unknown. In the current study, we demonstrate that PHB silencing results in mitochondrial fragmentation and adipogenic reduction in 3T3-L1 cells, uncovering for the first time a role for PHBs in mammalian adipogenesis.

## Materials and Methods

### Cell culture and adipocyte differentiation

The murine preadipocyte 3T3-L1 cell line was obtained from the American Type Culture Collection (ATCC; Manassas, VA) and cultured according to the manufacturer's instructions. Briefly, 3T3-L1 cells were cultured in growth medium, consisting of Dulbecco's Modified Eagle's Medium (DMEM) (Invitrogen; Carlsbad, CA) supplemented with 10% cattle bovine serum (CBS), 100 U/ml penicillin and 100 ug/ml streptomycin. The cultures were maintained in a humidified atmosphere of 5% CO_2_ and 95% air at 37°C. The medium was replaced every 2–3 days. Adipocyte differentiation of 3T3-L1 cells was induced by using standard hormonal cocktail. In brief, at two days post-confluence (day0), cells were treated with a differentiation medium containing DMEM supplemented with 10% fetal bovine serum (FBS), 0.25 µmol/L dexamethasone (Sigma; St. Louis, MO), 5 µg/ml insulin (Sigma) and 0.5 mmol/L isobutyl-methylxanthine (IBMX) (Sigma). At day 2, the medium was replaced with adipogenic medium containing DMEM supplemented with 10% FBS and 5 µg/ml insulin, which was changed every two days thereafter until analysis.

Human adipose tissue derived stem cells (ASC) and their culture medium were purchased from Invitrogen and cultured according to the manufacturer's manual. Briefly, human ASC were cultured in ASC growth medium containing basal medium, growth supplement and 2 mmol/L L-glutamine. The cultures were maintained in a humidified atmosphere of 5% CO_2_ and 95% air at 37°C. The medium was replaced every 2–3 days. Passages 5–6 were used for all experiments. To initiate differentiation, two days post-confluent ASC (day0) were treated with a differentiation medium containing ASC basal medium, 10% FBS, 2 mmol/L L-glutamine, 1 µmol/L dexamethasone, 10 µmol/L insulin, 0.5 mmol/L IBMX, 200 µmol/L Indomethacin (Sigma), 100 U/ml penicillin and 100 ug/ml streptomycin. The differentiation medium was changed every three days thereafter until the indicated times.

### Immunoblotting

Cells were lysed in mammalian protein extraction reagent (Thermo Scientific) supplemented with protease inhibitor cocktail (Sigma). Additionally, phosphatase inhibitor cocktail I and II were added for phospho-ERK (p-ERK) detection. The cell lysates were resolved by electrophoresis on 10% or 4–12% precast Bis-Tris gel (Invitrogen). Proteins were transferred from the gel to a nitrocellulose membrane using the iBlot Dry Blotting System (Invitrogen). Specific proteins were detected by immunoblotting using primary antibodies anti-PHB1 (BioLegend, San Diego, CA ), anti-PHB2 (Bethyl, Montgomery, TX), anti-C/EBPβ (Cell Signaling; Danvers, MA), anti-PPARγ (Cell Signaling), anti-aP2 (Abcam; Cambridge, MA), anti-HSP90 (Santa Cruz Biotechnology; Santa Cruz, CA) anti-β-actin (Santa Cruz), anti-ERK (Cell Signaling), anti-p-ERK (Cell Signaling) and anti-porin (Abcam). Horseradish peroxidase (HRP)-conjugated anti-rabbit IgG and anti-mouse IgG were used as secondary antibodies (Jackson ImmunoResearch; West Grove, PA). Blots were revealed with enhanced chemiluminescent reagents (Thermo Scientific).

### Real-time PCR analysis

Real-time PCR analysis was performed as previously described with slight modifications [28]. Briefly, total RNA was isolated from 3T3-L1 cells at the indicated times using the RNeasy Mini Kit (Qiagen; Valencia, CA). Isolated RNA was reverse-transcribed with oligo-dT primer using the Advantage RT-for-PCR kit (Clontech; Mountain View, CA). Real-time PCR was performed using the LightCycler FastStart DNA Master SYBR Green I kit (Roche Diagnostics; Indianapolis, IN) and a LightCycler real-time thermal cycler (Roche Diagnostics). The primer pairs used were: 5′-CCGTGGCGTACAGGACATTG-3′ and 5′- AAGGCAGGACCCGCTCATCATAGT-3′ for mouse PHB1; 5′- CGTGGAAGGCGGTCATAGAGC-3′ and 5′-TCGGGACAGCACACGCAGGGAGAT-3′ for mouse PHB2; 5′-GGAAGGGCACCACCAGGAGT-3′ and 5′-TGCAGCCCCGGACATCTAAG-3′ for mouse 18S rRNA as an internal control. The amplified products were analyzed by electrophoresis on 2% agarose gels containing ethidium bromide (E-Gels; Invitrogen) to confirm primer specificity and PCR product size.

### Transfection of siRNA in 3T3-L1 cells

One day before transfection, 3T3-L1 cells were seeded in the growth medium without antibiotics so that they would be 50–70% confluent at the time of transfection. Cells were transfected with 10 nmol/L siRNA using Lipofectamine RNAiMAX, according to the manufacturer's protocol (Invitrogen). The three siRNAs, siPHB1-1, siPHB1-2 and siPHB1-3, target the following distinct sequences in mouse PHB1 mRNA: 5′-GCUGUCAUCUUUGACCGAUTT-3′, 5′-GCUUCCUCGUAUCUACACCTT-3′, and 5′-GCCGCUGUCAAUAAAUGACTT-3′, respectively. The three siRNAs, siPHB2-1, siPHB2-2 and siPHB2-3, target the following distinct sequences in mouse PHB2 mRNA: 5′-GCACUGAGCAAGAAUCCUGTT-3′, 5′-GCAAGAAUCCUGGCUAUAUTT-3′ and 5′-GGCAUCAUGUGAUGGACUUTT-3′, respectively. A universal siRNA Control (siControl) was used as the negative control. All siRNAs were obtained from Invitrogen.

### Oil Red O staining

Oil red O (Sigma) staining was performed as suggested by the manufacturer with minor modifications. Seven days after the induction of adipocyte differentiation, 3T3-L1 cells in 60 mm dishes were washed with PBS and fixed with 10% formalin. The dishes were washed once with 60% isopropanol and left to dry completely. The cells were then stained in 0.2% Oil Red O for 10 minutes, rinsed with 60% isopropanol once, and thoroughly washed with water four times. The dishes were subsequently scanned to get the pictures. After extracting the Oil Red O with 100% isopropanol, the extracted dye was quantified on a spectrophotometer (Molecular Devices; Sunnyvale, CA) by reading the absorbance at 510 nm wave length.

### Immunocytochemistry

The procedure used for immunofluorescence microscopy of cells to detect PHBs has been previously described in detail [19], [20]. In brief, cover glasses were autoclaved and coated with attachment factor solution (Cell Applications; San Diego, CA) in a 6-well plate. 3T3-L1 cells were seeded and treated with differentiation medium in the plate as described above. The cells were then fixed with 4% paraformaldehyde in PBS for 30 min, followed by PBS wash and subsequent treatment with cold absolute methanol for 5 min at −20°C. The cells were rinsed with PBS and permeabilized with 0.2% Triton X-100 in PBS for 10 min. After blocking with 5% BSA in PBS for 1 hour, the cover glasses were incubated with anti-PHB1 (Abcam, 1∶100), anti-PHB2 (Abcam, 1∶50) or anti-Cytochrome C (CytC) (Invitrogen, 1∶500) antibody in 0.1% BSA in PBS at room temperature for two hours. The cells were then washed with PBS and incubated with Rhodamine (Invitrogen, 1∶250) or Alexa Fluor 488 (Invitrogen, 1∶500) conjugated secondary antibody in 0.1% BSA in PBS at room temperature for 1 hour. Thereafter, the cover glasses were mounted upside down on microscope slides containing mounting medium (Vector Laboratories; Burlingame, CA). The mounted slides were examined under an Olympus BX41 microscope equipped with an Optronics Magnafire digital camera and Prior Proscan motorized driven stage (Olympus, Melville, NY). For each image, specific antibody staining was merged with CytC (red) using Soft Imaging System software that results in virtually no pixel shifting during the image merge. Representative photomicrographs were arranged using Adobe Photoshop without any further adjustment to maintain the true nature of the findings.

### Isolation of mitochondria

Isolation of mitochondria was performed using a mitochondria isolation kit (Thermo Scientific) according to the manufacturer's protocol and previous descriptions [19], [20]. Briefly, pre- and seven days post-adipocyte differentiation 3T3-L1 cells in 100 mm dishes were harvested using trypsinization. A reagent-based method was used for cell homogenization allowing multiple samples to be processed concurrently. To obtain a more purified fraction of mitochondria with more than 50% reduction of lysosomal and peroxisomal contaminants, the last step for pelleting mitochondria was performed using centrifugation for 15 minutes at 3,000× *g* rather than at standard 12,000× *g*.

### Detection of mitochondrial DNA content

Total DNA in 3T3-L1 cells was isolated with the DNeasy DNA isolation kit (Qiagen). The DNA levels of mitochondrial Complex I and nuclear 18S rRNA were determined by real-time PCR quantification. The relative mtDNA content was evaluated by the ratio of DNA levels between mitochondrial Complex I and nuclear 18S rRNA as previously described [29].

### Transmission electron microscopy (TEM)

Three days post transfection of siRNA, 3T3-L1 cells were fixed with 2.5% glutaraldehyde in 0.1 mol/L sodium cacodylate buffer for two hours at 4°C, and post-fixed with 1% osmium tetroxide in 0.1 mol/L sodium cacodylate buffer for one hour at 4°C. The specimens were then incubated in 0.5% aqueous uranyl acetate for 2 hours at room temperature for *en bloc* staining, and in a graded series of ethanol for dehydration. Thereafter, the specimens were embedded in Embed 812 resin. Ultrathin sections were cut and post-stained with uranyl acetate and lead citrate. These sections were examined using a JEOL 1200EX transmission electron microscope (Tokyo, Japan).

### Mitotracker staining and confocal microscopy

MitoTracker Red CMXRos (Invitrogen), a mitochondria-specific cationic fluorescent dye, was used to label mitochondria. 3T3-L1 cells in Lab-Tek chamber slides (Thermo Scientific) were stained with 250 nmol/L MitoTracker in serum-free DMEM for 15 minutes at 37°C according to the manufacturer's instructions. A Leica TCS SP5 Confocal Microscopy System (Leica Microsystems; Bannockburn, IL) equipped with a 63×/1.40 NA oil-immersion objective lens was used to characterize the optical properties of these samples. Images were captured with a scanning speed of 400 Hz and image resolution of 512×512 pixels, and then analyzed using Leica Application Suite, Advanced Fluorescence (LAS AF) software.

### Measurement of ATP concentration

The adenosine triphosphate (ATP) concentration was measured with an ENLITEN ATP assay system bioluminescence detection kit (Promega; Madison, WI). Briefly, three days after transfection of 3T3-L1 cells in a 96-well plate with siRNA oligonucleotides, 0.5% trichloroacetic acid (TCA) was added for ATP efficient release. Then, 25 mmol/L Tris-acetate (pH 7.75) was used for neutralization. After addition of recombinant Luciferase/Luciferin reagent (rL/L), luminescence was measured using a 10-second integration time with a microplate luminometer (Lmax) and SOFTmax PRO software (Molecular Devices), and was normalized to protein concentration. The ATP standard curve was generated by using the ATP standard (10^−7^ mol/L) included in the kit.

### Reactive oxygen species (ROS) detection

ROS were detected with the cell-permeable, peroxide-sensitive fluorophore, 2′,7′-dichlorofluorescein diacetate (DCF-DA) (Sigma). In brief, 3T3-L1 cells in a 6-well plate were incubated in 0.2 µmol/L DCF-DA at 37°C for 30 minutes. Cells were then washed with prewarmed PBS twice and allowed to recover in growth medium for 20 minutes at 37°C in an atmosphere of 5% CO_2_. Afterwards, the cells were trypsinized, washed, resuspended in PBS and analyzed by running ExpressPlus assay on a flow cytometer, Guava PCA-96 AFP (Millipore; Billerica, MA). To ensure that DCF-DA was detecting hydrogen peroxide, cells were preincubated with 250 U/ml cell-permeable polyethylene glycol (PEG)-catalase (Sigma) at 37°C for two hours.

### Detection of mitochondrial complex I activity

The activity of complex I was determined in whole cell lysates of 3T3-L1 with the Mitochondrial Dipstick Assay kit according to the manufacturer's instructions (Abcam). Twenty-five micrograms of proteins were allowed to wick up through the dipstick membrane. The dipsticks, with complex I immunocaptured, were transferred into the complex I enzyme substrate buffer. The enzyme activities were then calculated by measuring the optical density of precipitation, colorimetric enzyme reaction products, using the NIH ImageJ software. Standard curves were created from multiple determinations of complex activities in cultured 3T3-L1 cell extracts.

### Statistics

All samples were at least prepared in triplicate. Results from the quantitative studies are expressed in terms of mean and standard deviation (mean ± SD) of three independent experiments. Statistical analyses were performed by one-way ANOVA and comparisons between groups were performed using the Student's *t* test. Differences were considered significant at *p*<0.05.

## Results

### PHBs are up-regulated during adipogenesis of mouse and human preadipocytes

**Figure 1:**
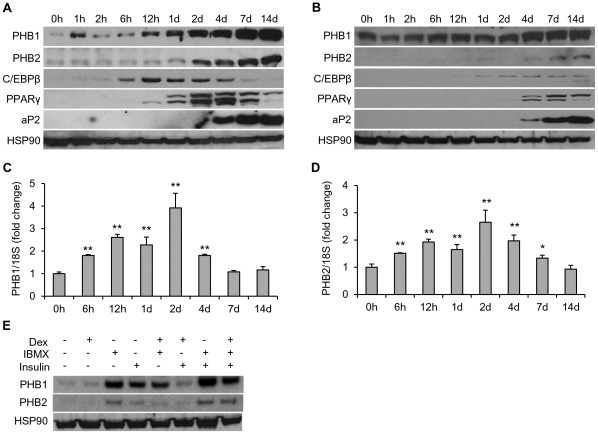
Expression of PHBs during adipogenesis. Over-confluent 3T3-L1 preadipocytes (**A, C, D**) or human ASC (**B**) were treated with the standard adipocyte-differentiation medium for indicated times. **A and B.** The protein levels of PHBs and adipogenic markers, C/EBPβ, PPARγ and aP2, were analyzed by immunoblotting. HSP90 was used as loading control. h, hours; d, days. **C and D.** The relative mRNA expression levels of PHB1 (**C**) and PHB2 (**D**) were analyzed by real-time PCR. 18S rRNA was used as an internal control. * *p*<0.05, ** *p*<0.01 compared to 0 h. **E**. 3T3-L1 cells were treated with DMEM supplemented with 10% FBS and a different combination of dexamethasone (Dex), insulin and IBMX as indicated for 24 hours. The expression levels of PHB1 and PHB2 proteins were analyzed by using Immunoblotting. HSP90 was used as the loading control.

In the 3T3-L1 adipogenic model, we observed that the protein levels of both PHB1 and PHB2 continuously increased during the entire 14 days of adipocyte differentiation (Fig. 1A), consistent with a prior report of a microarray analysis showing that the expression levels of PHBs are increased during 3T3-L1 cell adipogenesis [25]. The sequentially induction of the adipogenic markers, CCAAT/enhancer-binding protein beta (C/EBPβ), peroxisome proliferator-activated receptor gamma (PPARγ) and adipocyte Protein 2 (aP2), were observed in these conditions. Additionally, we determined that the alterations of protein levels of PHB1 and PHB2 followed a similar pattern during adipogenesis in human ASC compared to mouse 3T3-L1 cells (Fig. 1B). When we examined the mRNA levels of PHBs in differentiating 3T3-L1 cells, we found that both PHB1 and PHB2 were significantly increased as early as six hours post adipogenic induction and peaked at day two (Fig. 1C and 1D) and fell to basal levels by one to two weeks, suggesting post-translational protein stabilization. Among the three hormone ingredients in the adipogenic cocktail, the PHBs expression was mainly induced by IBMX and insulin rather than dexamethasone in 3T3-L1 cells (Fig. 1E). In addition, the levels of PHBs in white adipose tissue (WAT) from wild-type, heterozygous and homozygous obese mice, both female and male, were compared. Interestingly, the expression levels of PHBs in WAT from obese mice were not higher than that from wild type ones (Fig. S1). Actually, even decrease of the expression of adipogenic genes in obesity has been observed [30], which is considered as the consequence of the dedifferentiation in WAT from obese mice.

### PHBs are required for PPARγ expression and adipocyte differentiation in 3T3-L1 cells

**Figure 2:**
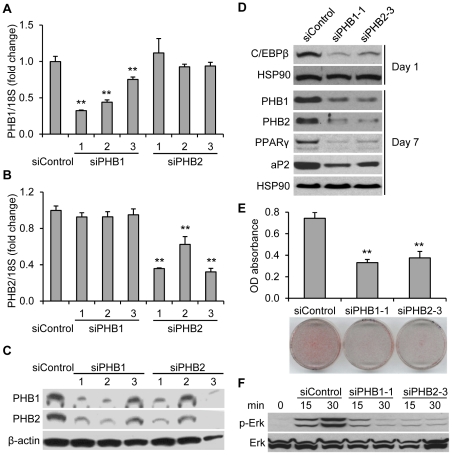
Silencing of PHBs reduced the adipogenesis in 3T3-L1 cells. **A, B and C.** 3T3-L1 preadipocytes were transfected with the siControl or siRNAs targeting PHB1 (siPHB1-1, siPHB1-2 and siPHB1-3) or PHB2 (siPHB2-1, siPHB2-2 and siPHB2-3), respectively. **A and B.** Cells were harvested for total RNA isolation and quantitative real-time PCR to determine the mRNA expression levels of PHB1 (**A**) and PHB2 (**B**) three days post transfection. 18S rRNA was used as an internal control. ** *p*<0.01 compared to siControl. **C.** Cells were harvested to determine the protein expression levels of PHB1 and PHB2 with Immunoblotting three days post transfection. β-actin was used as a loading control. **D, E and F.** 3T3-L1 cells were treated with adipogenic inducers (day 0) three days post transfection of siRNAs targeting PHB1or PHB2. siControl was used as the control for siPHBs. **D.** The levels of C/EBPβ at day1, and the levels of PHB1, PHB2, PPARγ and aP2 at day 7 were determined using immunoblotting. HSP90 was used as a loading control. **E.** The cells were stained with Oil Red O dye at day 7. The quantification of accumulated lipid was performed by readings on a spectrophotometer at 510 nm for cell-released dye. **p<0.01 compared to siControl. **F.** Phospho-ERK (p-ERK) in siRNA transfected 3T3-L1 cells was detected using immunoblotting at the indicated times of treatment with adipogenic inducer cocktail. ERK was used as a loading control.

To investigate the possible roles of increasing PHBs during adipogenesis, we screened siRNA for effective knockdown of PHBs expression in this model. Three different siRNA oligonucleotides, siPHB1-1, siPHB1-2 and siPHB1-3, were used to target PHB1; while siPHB2-1, siPHB2-2 and siPHB2-3 were used to target PHB2. Commercially available universal siControl was used as a control. Three days after the transfection of siRNA in 3T3-L1 cells, the mRNA levels of PHB1 were markedly reduced by siPHB1-1 (68%), siPHB1-2 (56%) or siPHB1-3 (25%), whereas the mRNA levels of PHB2 were not significantly changed (Fig. 2A). Similarly, the mRNA levels of PHB2 were decreased by siPHB2-1 (64%), siPHB2-2 (38%) or siPHB2-3 (68%), whereas mRNA levels of PHB1 were virtually identical (Fig. 2B). Interestingly, the protein levels of both PHB1 and PHB2 could be down-regulated by either siPHB1 or siPHB2 transfection (Fig. 2C). These data suggest that, in mouse 3T3-L1 preadipocytes, PHB1 and PHB2 are present in an interdependent complex and are consistent with previous findings in yeast, *C. elegans*, MEFs, human HeLa cells and MCF-7 cells [12], [14], [16], [17], [18]. Our earlier studies showed that PHB silencing could increase cellular sensitivity to apoptosis [20]. In the current study, we did not see the induction of cleaved caspase-3 in 3T3-L1 cells by simply silencing PHB1 or PHB2 without an apoptotic inducer (data not shown), thus consistent with the observation in PHB2-deficient MEFs [18]. For all of the following experiments, siPHB1-1 and siPHB2-3 were used to knockdown PHB1 and PHB2, respectively.

Using the loss-of-function strategy, our results demonstrated that expression levels of the adipogenic markers, C/EBPβ at day 1, PPARγ and aP2 at day 7 (Fig. 2D), and the accumulation of lipids (Fig. 2E) were significantly decreased after adipogenic induction in PHB1- or PHB2-silenced 3T3-L1 cells. Additionally, based on the reports that PHB is required for Ras-induced Raf-MEK-ERK pathway activation in epithelial cells [31] and that activation of MEK/ERK signaling is necessary to initiate the preadipocyte in the differentiation process during the early phase [32], [33], we determined the level of phosphorylated ERK1/2 at the early stage of adipogenic induction upon PHB-silencing in 3T3-L1 cells. Our data demonstrated that the phosphorylation of ERK1/2 is remarkably inhibited post-adipogenic induction in either PHB1 or PHB2-silencing 3T3-L1 cells (Fig. 2F), suggesting that the effect of PHBs in adipogenesis might be *via* ERK phosphorylation in 3T3-L1 cells.

Interestingly, when using the gain-of-function strategy, a slight decrease in adipocyte markers and lipid accumulation (80% of control) were observed upon overexpression of PHB1 in adipocyte-differentiating human ASC (Fig. S2). These observations were in agreement with a recent report in 3T3-L1 cells with uncertain mechanisms [34].

### Enhanced levels of mitochondrial PHBs and mtDNA in adipogenic 3T3-L1 cells

**Figure 3:**
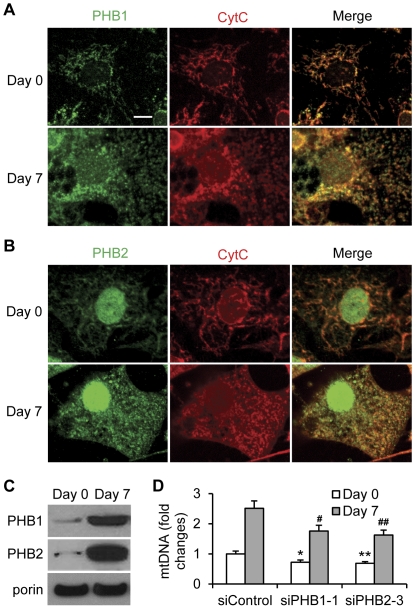
Content of mitochondrial PHBs and mtDNA in 3T3-L1 cells pre- and post-adipogenesis. **A, B and C.** Over-confluent 3T3-L1 cells (Day 0) were induced for adipocyte differentiation for 7 days. **A,** PHB1 (green) or **B,** PHB2 (green) was detected by using immunocytochemistry. CytC (red) was used as the mitochondrial marker. Bar = 10 µm. **C.** The levels of PHB1 and PHB2 in isolated mitochondria were detected using the immunoblotting analysis. The mitochondrial marker porin, also called the voltage dependent anion channel (VDAC), was used as a loading control. **D.** 3T3-L1 cells were transfected with indicated siRNAs and cultured for three days. The relative mtDNA content was evaluated by a ratio of the DNA level of mitochondrial Complex I to the DNA level of nuclear 18SrRNA. The relative mtDNA content in 3T3-L1 cells transfected with siControl was set to 1. *p<0.05, **p<0.01 compared to siControl (Day 0). ^#^p<0.05, ^##^p<0.01 compared to siControl (Day 7).

The essential role of mitochondria in adipocyte differentiation is well described [4], [5], [6], [7], [35], which led us to examine the changes in the levels of mitochondrial PHBs before and after 3T3-L1 cell adipogenesis. Our immunocytochemistry results showed that either PHB1 or PHB2 in mitochondria was increased after seven days of adipocyte differentiation (Fig. 3A and 3B). PHB1 were mainly present in mitochondria while PHB2 in mitochondria and nuclei. The contents of PHBs were further determined in isolated mitochondria and nuclei. Our data demonstrated that the protein levels of PHB1 and PHB2 were remarkably increased in mitochondrial fraction (Fig. 3C) and slightly increased in nuclear fraction (Fig. S3), seven days post adipogenic induction. These findings indicate that the recruitment of PHBs to the mitochondria is enhanced during adipogenesis and that this is not simply because of increased mitochondrial biogenesis. To investigate the effects of PHBs on mitochondrial biogenesis during adipogenesis, the relative contents of mitochondrial mtDNA were examined in 3T3-L1 cells upon PHB knockdown and adipocyte-differentiation induction. Our results showed that the contents of relative mtDNA were significantly increased in 3T3-L1 cells subject to adipogenic induction. The increments of mtDNA were partially suppressed upon PHB1- or PHB2- silencing in 3T3-L1 cells, whether or not subject to adipogenic induction (Fig. 3D). These results, in accordance with the observations in HeLa cells [21], suggest that PHBs are required in maintaining mitochondrial contents.

### Effects of PHB silencing on mitochondria in 3T3-L1 cells

**Figure 4:**
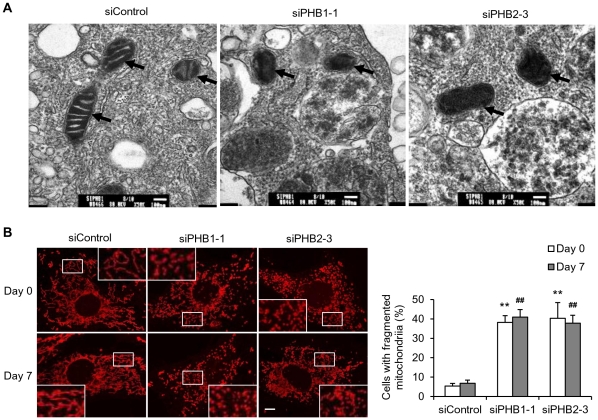
Effect of PHB knockdown on the morphology of mitochondria in 3T3-L1 cells. 3T3-L1 cells were transfected with siRNA targeting PHB1 or PHB2, and cultured for three days. siControl was used as the control. **A.** The cristae morphology of mitochondria (arrows) in 3T3-L1 cells, transfected with the indicated siRNAs, was assessed by transmission electron microscopy (TEM) at 80 kV acceleration voltage and 50 k magnification. Scale bar, 100 nm. **B.** The siRNA-transfected 3T3-L1 cells were subject to adipogenic induction for 7 days. The cells at day 0 and day 7 were stained with MitoTracker Red. The mitochondrial morphology was analyzed with a Confocal Microscopy System. Scale bar, 10 µm. High-power magnification insets are included to emphasize the comparison of tubular mitochondria to fragmented ones. The bar graph represents the percentage of cell populations with fragmented mitochondria. **p<0.01 compared to siControl at day 0; ^##^ *p*<0.01 compared to siControl at day 7.

The ultrastructure of mitochondria in siRNA transfected 3T3-L1 cells was examined using electron microscopy. We observed that the regular lamellar cristae in mitochondria were lost in siPHB1- or siPHB2-transfected 3T3-L1 cells, whereas siControl transfection did not affect the ultrastructure of mitochondria (Fig. 4A). To further investigate the effects of mitochondrial PHBs during adipogenesis, the mitochondrial morphology of PHB-silencing 3T3-L1 cells was compared before and after adipogenic induction. MitoTracker analysis revealed that, instead of normally tubular mitochondria, about 40% of the PHB1 or PHB2 knockdown cells consisted of fragmented mitochondria either before or after the cellular adipogenesis (Fig. 4B). Actually, punctiform mitochondria have been observed in our previous study upon PHB1-knockdown in ovarian cancer cells [20].

**Figure 5:**
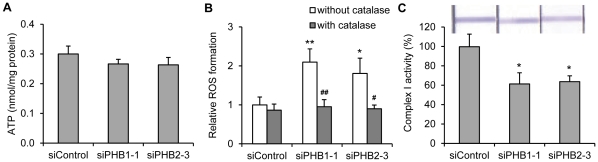
PHB deficiency induces the accumulation of ROS without affecting the formation of ATP. 3T3-L1 cells were transfected with indicated siRNAs and cultured for three days. **A.** ATP concentration was detected using a luminescence assay, and was normalized to protein concentration. **B.** Cells were preincubated with/without 250 U/ml PEG-catalase for two hours. ROS formation was detected by using DCFA-DA staining and flow cytometry. *p<0.05, **p<0.01 compared to siControl without PEG-catalase preincubation; ^#^ *p*<0.05 compared to siPHB2-3 without catalase preincubation; ^##^ *p*<0.01 compared to siPHB1-1 without catalase preincubation. **C.** Activities of complexes I were determined in whole cell lysates of 3T3-L1 with the Mitochondrial Dipstick Assay kit. The Complex I activity in 3T3-L1 cells transfected with siControl was set to 100%. *p<0.05, compared to siControl.

Because a lack of PHB1 or PHB2 might reduce mitochondrial membrane integrity, disrupt OXPHOS and therefore result in increased levels of ROS [21], [23], [36], [37], we examined the ATP accumulation and ROS formation upon PHB1- or PHB2-silencing in 3T3-L1 preadipocytes. Our results showed that ROS levels were significantly increased in either PHB1- or PHB2-knockdown 3T3-L1 cells, whereas the contents of ATP were unaffected. The increase of ROS could be ablated when the cells were preincubated with PEG-catalase, a hydrogen peroxide scavenger, indicating the specificity of the DCF-DA signal for hydrogen peroxide (Fig. 5A and 5B). These results, in agreement with the effects of PHB deficiency on wild type *C. elegans* [27], suggest the damage of mitochondrial OXPHOS system upon PHB-silencing in 3T3-L1 cells. To further investigate the underlying mechanisms of the extra ROS generation, mitochondrial complex I activity was examined. Our data demonstrated a reduction of the complex I activity in PHB1- or PHB2-knockdown 3T3-L1 cells (Fig. 5C). This result is in accordance with the observation in the PHB1-depleted endothelial cells [23], indicating the affection of mitochondrial electron transport in the OXPHOS system.

## Discussion

It is well established and reviewed that mitochondrial biogenesis is essential during adipocyte differentiation; and that PHBs complexes, located in the inner membrane of mitochondria, play a crucial role in mitochondrial morphology and dynamics [22], [35], [38], [39]. A recent study provides the evidence that depletion of PHB1 or PHB2 in *C. elegans* significantly decreases mitochondrial membrane potential and adipose accumulation in young adults [27]. However, studies on the effects of PHBs in mammalian adipogenesis are currently lacking. Data presented here indicate that the levels of PHBs are remarkably increased during adipogenesis in 3T3-L1 cells, and silencing of PHBs causes mitochondrial fragmentation and adipogenic reduction.

Either PHB1 or PHB2-knockout mice exhibit early embryonic lethality, indicating that these proteins have fundamentally important functions [14], [40]. In primary mouse adipocytes, PHB1 decreases insulin-stimulated oxidation of glucose and fatty acid, implying that PHB1 may play a role in promoting fat accumulation [41]. Indeed, our results showed the incremental mRNA and protein expression of PHBs in a time course manner using real-time PCR and immunoblotting, which is consistent with the prior observations that intracellular expression of PHBs is increased and extracellular secretion of PHBs is decreased during 3T3-L1 adipocyte differentiation upon genetic and proteomic approaches [25], [26]. In addition, we observed that the PHBs expression was mainly induced by insulin and IBMX rather than glucocorticoid among the three components in adipogenic induction cocktail. Insulin is known to act through the insulin-like growth factor 1 (IGF-1) receptor [42]. Stimulation of the IGF-1 receptor regulates cMyc [43], which is reported to be a transcription factor of PHB [44]. IBMX, a cyclic adenosine monophosphate (cAMP) phosphodiesterase inhibitor, prevents the inactivation of the intracellular cAMP, and therefore enhances expression of C/EBPβ, a critical transcription factor at the early stage of adipogenic program [45]. Taken together, we postulate that the induction of PHBs is probably initiated *via* IGF1, cMyc and/or cAMP molecules. Interestingly, we further observed that the expression of PHBs in WAT from obese mice is not more than that from lean ones. In fact, the decrease of the genes, that are characteristic of mature adipocytes and transcription factors critical to the maintenance of terminally differentiated fat cells, are reported in WAT of obese mice. This implies that some degree of dedifferentiation has taken place in the adipose tissue of obese mice [30].

PHB1 and PHB2 are highly homologous proteins that are evolutionarily conserved and ubiquitously expressed [46], [47]. A study in yeast has initially shown that PHB1 and PHB2 act as mitochondrial chaperones in the inner mitochondrial membrane [48]. The interdependence of both PHBs was subsequently reported in nematode [12] and some types of mammalian cells by several independent groups including ours [14], [17], [18], [49]. To study the function of PHBs in 3T3-L1 cells, we employed a loss-of-function strategy and found that the loss of one simultaneously leads to the loss of the other at the protein level. Upon silencing of the PHB1 or PHB2, we observed a lower degree of fat accumulation in adipogenic 3T3-L1 cells. Indeed, a recent observation has shown that PHB deficiency markedly reduces intestine fat content early in adulthood of wild-type nematodes. Interestingly, in both nhr-49 and fat-7 mutant nematodes, which causes fat accumulation due to decreased synthesis of monounsaturated fatty acids, deficiency of PHB not only reduces intestinal fat but also prevents shortage of lifespan [27]. Since either the PHB1- or PHB2-conventional knockout mice do not survive, adipocyte-specific PHB conditional knockout mice may be used in future adipogenic studies. Besides fat accumulation, we detected a downregulation of the adipogenic markers, C/EBPβ at the early stage and the PPARγ and aP2 at the late stage, upon silencing of PHBs in 3T3-L1 cells, which confirms the essential role of PHBs during adipogenesis. This also implies that PPARγ, a key molecule in adipogenesis, may be located downstream of PHBs during adipocyte differentiation. Interestingly, upon forced expression of PHB1 in human ASC, our data demonstrated that the adipocyte differentiation was reduced rather than enhanced. This result is in line with a recent report that adipogenesis is inhibited in 3T3-L1 cells. However, in the absence of insulin, overexpression of PHB1 facilitates adipogenesis of 3T3-L1 cells after adipogenic initiation with adipocyte-induction cocktail. This may be one of the underlying mechanisms involved in enhanced adipogenesis under insulin-resistance condition [34]. It will be interesting to determine the effects of insulin-lacking adipocyte-induction cocktail on adipogenesis and mitochondrial biology in human ASC upon overexpression of PHB1.

PHB plays an important role in the Ras-mediated activation of the Raf/MEK/ERK pathway [31], [50], which is a highly conserved signaling module that regulates a multitude of essential cellular functions such as proliferation and differentiation [51]. In addition, the activation of MEK/ERK signaling promotes adipogenesis by enhancing PPARγ and C/EBPα gene expression during the early phase of the differentiation of 3T3-L1 preadipocytes [32], [33]. Our data, in agreement with the above observations, further demonstrate that PHBs are required for the phosphorylation of ERK as early as 15 minutes post adipogenic induction in 3T3-L1 cells.

Mitochondrial biogenesis is essential in adipocyte differentiation. A 20- to 30-fold increase in the concentration of many mitochondrial proteins has been observed during adipogenesis in a proteomic analysis [4]. It is reported that inhibition of mitochondrial citrate export causes a significant reduction in fat accumulation in 3T3-L1 cells [6]. In addition, DNA binding of PPARγ induced by the adipogenic cocktail is completely prevented in preadipocytes treated with an inhibitor of mitochondrial respiration [7]. In the present study, we detected a remarkable increment of mitochondrial content and PHBs in differentiated adipocytes when compared to preadipocytes. Furthermore, in the mitochondrial fraction, the concentration of PHB1 or PHB2 is much higher in adipocyte-differentiated 3T3-L1 cells, suggesting that the increase of mitochondrial PHB1 or PHB2 is beyond the increase of the mitochondrial mass during adipogenesis. The mitochondrial content is reduced in PHB-deficient 3T3-L1 cells, which may be one of the mechanisms to explain our previous findings that PHB is essential for stabilizing the mitochondrial integrity and membrane potential [19], [20]. Interestingly, our data demonstrate that the protein levels of PHB1 and PHB2 in nuclear fractions are slightly increased in 3T3-L1 cells upon adipocyte differentiation. The mitochondrial-nuclear communication by PHBs shuttling under cell stress or cell differentiation has been recently reported [17], [52], [53]. However, the existence and influence of nuclear translocation of PHBs during 3T3-L1 cell adipogenesis remain unclear.

The analysis of the native structure of PHB1 and PHB2 in yeast, nematodes and mammals has revealed that both proteins are present in a high molecular-weight complex in the inner membrane of mitochondria [10], [12], [13]. The mitochondrial network is composed of highly interconnected tubules formed by balanced fusion and fission events [54]. Our previous study has shown that PHB1 down-regulation resulted in a transition of mitochondrial morphology from a normal reticular network to vesicular punctiform in ovarian cancer cells [20]. Here, we observed the loss of mitochondrial cristae and the fragmentation of mitochondrial network in either PHB1 or PHB2 knockdown 3T3-L1 preadipocytes. These findings are in agreement with the reports on PHB2-deficient MEFs and PHB1- or PHB2-silencing in HeLa cells [17], [18], which suggests that the fusion of mitochondrial membranes is impaired in the absence of PHBs. The abnormal mitochondrial morphology observed in the absence of PHBs may be explained by an altered processing of OPA1 [18], a large dynamin-like GTPase that is found in the mitochondrial intermembrane space and regulates both mitochondrial fusion and cristae morphogenesis [54]. The mechanism by which PHBs affect OPA1 processing remains to be determined.

Mitochondria are described as power plants because they generate most of the cellular supply of ATP, which is used as a source of chemical energy. We have not seen significant changes in ATP levels in 3T3-L1 preadipocytes upon PHB1- or PHB2-silencing. These observations are in accordance with the reports in PHB2-deficient MEFs and in PHB1- or PHB2-deficient wild-type *C. elegans* [18], [27]. Schleicher et al. has also reported that the degree of mitochondrial coupling of the respiratory chain in PHB1-knockdown endothelial cells was similar to the control cells [23]. In addition to their crucial role in energy homeostasis, mitochondria are the main site of ROS generation. Mitochondrial ROS have been proven to act as signaling molecules that impact many basic cellular functions such as cell differentiation. It has been demonstrated that mitochondrial ROS strongly inhibits adipocyte differentiation by specifically up-regulating C/EBPζ, a dominant-negative inhibitor which forms heterodimers with other C/EBP members [24]. By inhibiting adipogenesis, mitochondrial ROS may influence and limit the development of adipose tissue. Our data provide the evidence that the contents of ROS are enhanced in either PHB1 or PHB2-knockdown 3T3-L1 preadipocytes, which is consistent with the observation in PHB1-deficient endothelial cells and in PHB1- or PHB2- deficient nematodes [23], [27]. It is reported that the reason for the extra ROS generation may be the inhibition of mitochondrial complex I activity in PHB-depleted cells, and therefore affects mitochondrial electron transport in the OXPHOS system [22], [23]. Indeed, our results demonstrate a reduction of mitochondrial complex I activity in 3T3-L1 cells upon knockdown of PHB1 or PHB2. To maintain cytochrome oxidase activity and overall ATP production, there are compensatory mechanisms at play in mitochondria, involving an increase in electron flow through complex II and/or complex III, which may explain the unaffected ATP levels in this situation [23].

In summary, enhanced expression and mitochondrial recruitment of PHBs are required for maintaining mitochondrial morphology and inducing adipocyte differentiation in 3T3-L1 cells. These findings underscore the emerging concept of mitochondrial PHBs as important molecules in modulating fat metabolism [27]. Since both mitochondrial biogenesis and adipocyte differentiation have been linked to obesity, PHBs may become interesting candidates for further studies in this field.

## Supporting Information

Figure S1 **Expression of PHBs in mouse WAT.** The relative mRNA expression levels of PHB1 and PHB2 in WAT from male or female of wild type (ob^−/−^), heterozygous (ob^+/−^) and homozygous (ob^−/−^) obese mice were analyzed with real-time PCR. The materials and methods were described in Text S1. The 36B4 was used for normalization (n = 5).
(TIF)

Figure S2 **Effects of PHB1 overexpression on ASC adipogenesis.** Three days post transduction of Lenti/GFP (GFP) or Lenti/PHB1 (PHB1), ASC were treated with the adipocyte-differentiation medium for the indicated days. The materials and methods for creation and transduction of lentivirus were described in Text S1. **A.** The protein levels of PHB1 and adipogenic markers, C/EBPβ, PPARγ and aP2, were analyzed with immunoblotting. HSP90 was used as a loading control. d, days. **B.** The cells were stained with Oil Red O dye at day 14. The quantification of accumulated lipid was performed by readings on a spectrophotometer at 510 nm for cell-released dye. *p<0.05 compared to GFP.
(TIF)

Figure S3 **Content of nuclear PHBs in 3T3-L1 cells pre- and post-adipogenesis.** Over-confluent 3T3-L1 cells (Day 0) were induced for adipocyte differentiation for 7 days (Day 7). The levels of PHB1 and PHB2 in isolated nuclei were detected using immunoblotting analysis. The materials and methods for nuclear isolation were described in Text S1. The TBP was used as a loading control.
(TIF)

Text S1 **Supporting materials and methods.**
(DOC)
