# Supplementary figures and images for: Mitochondrial Dysfunction and Adipogenic Reduction by Prohibitin Silencing in 3T3-L1 Cells
Source: PLoS One. 2012 Mar 30;7(3):e34315. doi: 10.1371/journal.pone.0034315 (PMC3316679; doi:10.1371/journal.pone.0034315)

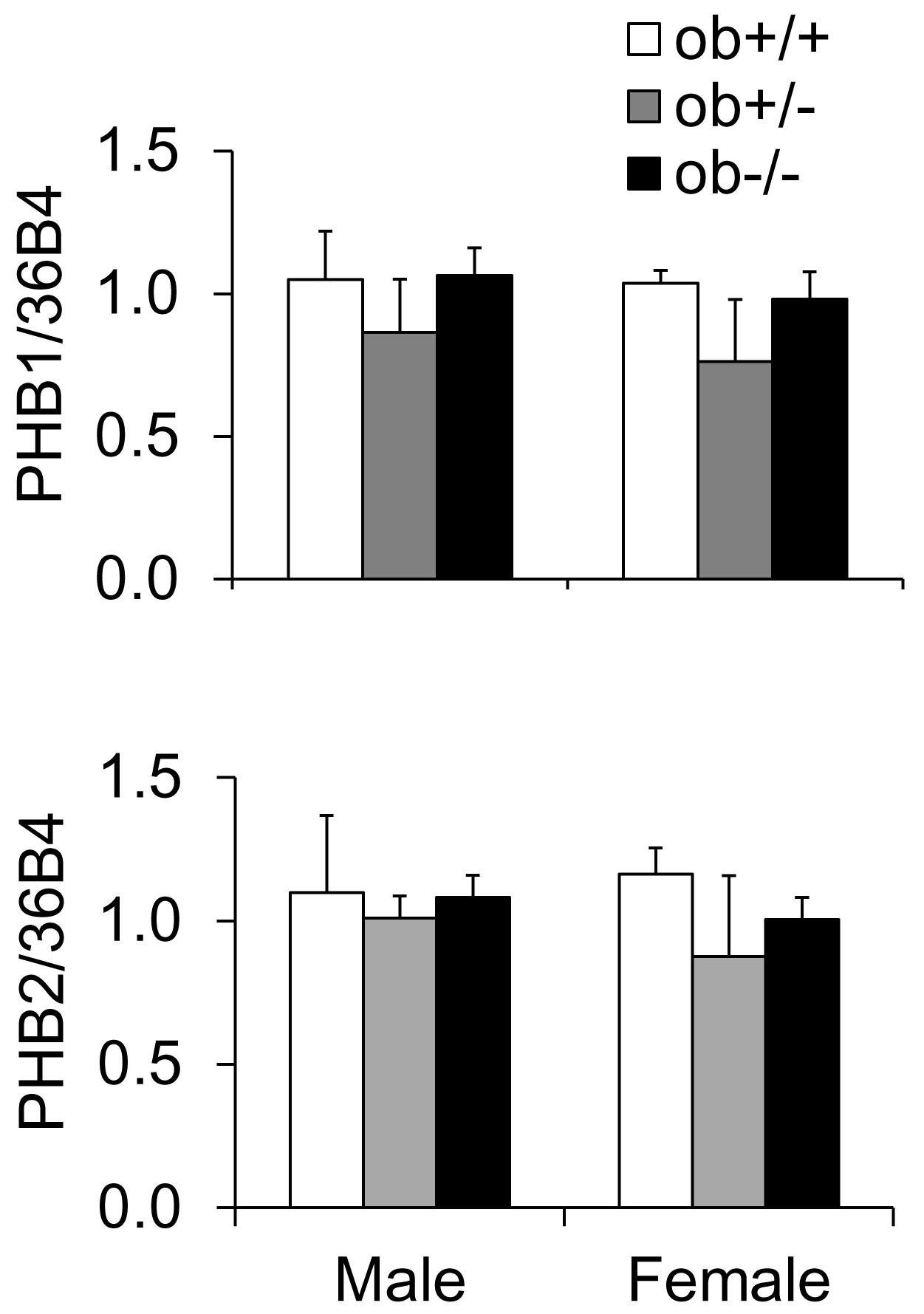

Supplement: Figure S1 — Expression of PHBs in mouse WAT. The relative mRNA expression levels of PHB1 and PHB2 in WAT from male or female of wild type (ob−/−), heterozygous (ob+/−) and homozygous (ob−/−) obese mice were analyzed with real-time PCR. The materials and methods were described in Text S1. The 36B4 was used for normalization (n = 5). (TIF) [file pone.0034315.s001.tif]

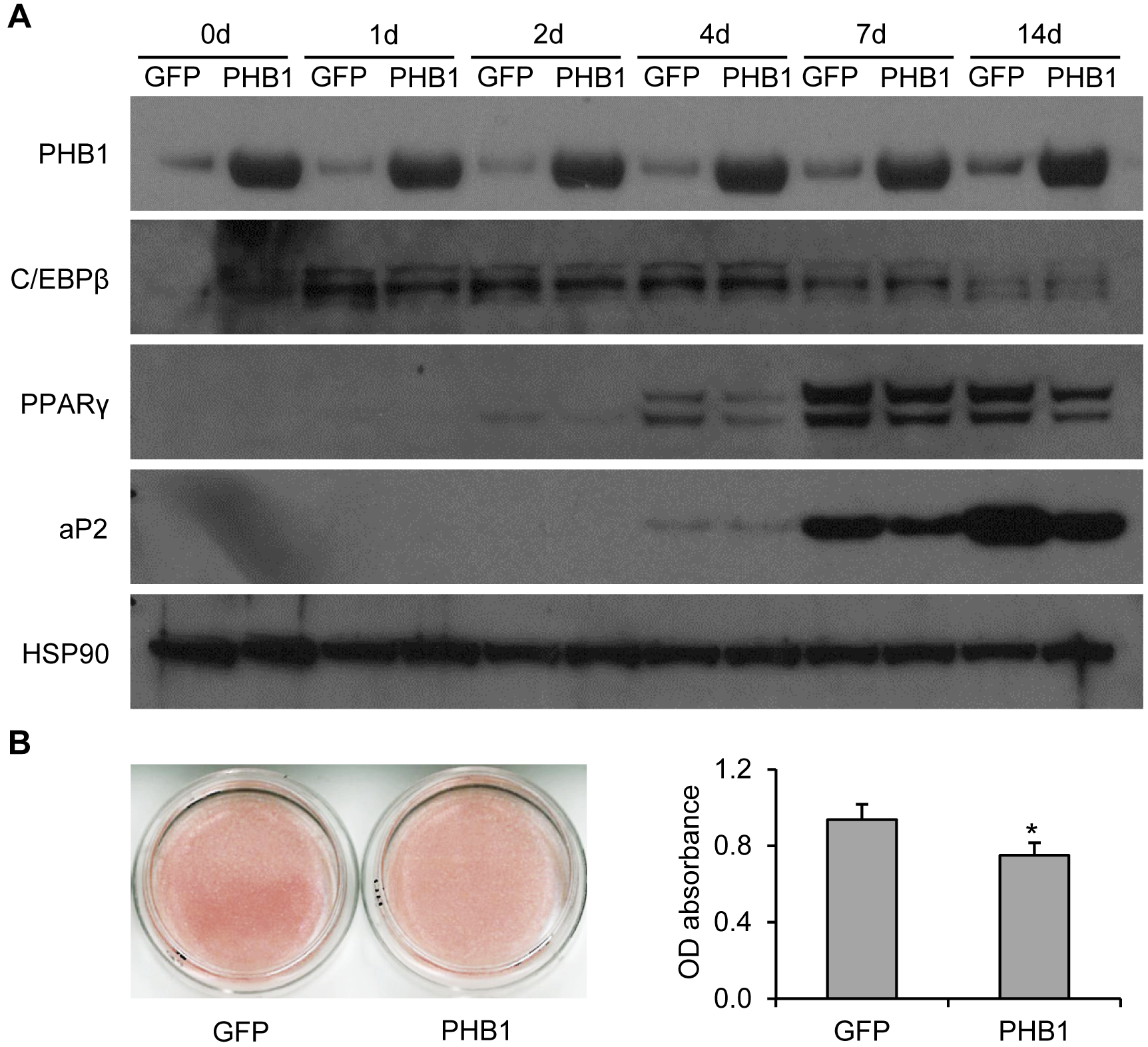

Supplement: Figure S2 — Effects of PHB1 overexpression on ASC adipogenesis. Three days post transduction of Lenti/GFP (GFP) or Lenti/PHB1 (PHB1), ASC were treated with the adipocyte-differentiation medium for the indicated days. The materials and methods for creation and transduction of lentivirus were described in Text S1. A. The protein levels of PHB1 and adipogenic markers, C/EBPβ, PPARγ and aP2, were analyzed with immunoblotting. HSP90 was used as a loading control. d, days. B. The cells were stained with Oil Red O dye at day 14. The quantification of accumulated lipid was performed by readings on a spectrophotometer at 510 nm for cell-released dye. *p<0.05 compared to GFP. (TIF) [file pone.0034315.s002.tif]

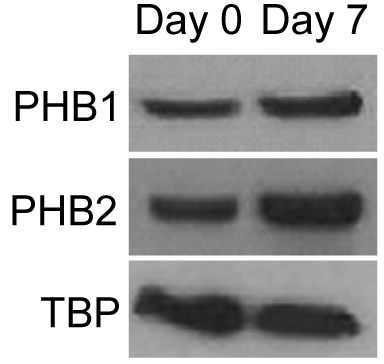

Supplement: Figure S3 — Content of nuclear PHBs in 3T3-L1 cells pre- and post-adipogenesis. Over-confluent 3T3-L1 cells (Day 0) were induced for adipocyte differentiation for 7 days (Day 7). The levels of PHB1 and PHB2 in isolated nuclei were detected using immunoblotting analysis. The materials and methods for nuclear isolation were described in Text S1. The TBP was used as a loading control. (TIF) [file pone.0034315.s003.tif]
