# Supplementary material for: Mitochondrial Dysfunction and Adipogenic Reduction by Prohibitin Silencing in 3T3-L1 Cells
Source: PLoS One. 2012 Mar 30;7(3):e34315. doi: 10.1371/journal.pone.0034315 (PMC3316679; doi:10.1371/journal.pone.0034315)
Supplement: Text S1 — Supporting materials and methods. (DOC) [file pone.0034315.s004.doc]

**SUPPORTING MATERIALS AND METHODS**

**Harvesting and real-time PCR analysis of mouse white adipose tissues**

Our study was approved by the Institutional Animal Care and Use Committee of the Atlanta University Center. Six weeks old, male and female of C57BL/6J, B6.V-Lep^ob+/-^/J and B6.V-Lep^ob-/-^/J mice were obtained from The Jackson Laboratory (Bar Harbor, ME) and were euthanized with CO_2_ inhalation. The mice were perfused with PBS by injecting from the left ventricle and flowing out from the opened right atrium. The white adipose tissues (WAT) from epididymal depot were harvested. Adipose tissues were homogenized using Lysing matrix D tubes and a FastPrep homogenizer (MP Biomedicals; Solon, OH) in RNA isolation buffer. The real-time PCR analysis was performed as described in the main ‘Materials and Methods’. The primer pair used for mouse 36B4 was: 5'-AAGCGCGTCCTGGCATTGTCT-3' and 5'-CCGCAGGGGCAGCAGTGGT-3'.

**Creation and transduction of Lenti/PHB in 3T3-L1 cells**

Total RNA from human ovarian cells was isolated and reverse-transcribed to cDNA as described above in ‘Real-time PCR analysis’ and our previous work . PHB1 overexpression plasmid, pLenti/PHB1, was created by employing Gateway technology (Invitrogen) as we did previously . Briefly, the PHB1 cDNA was amplified by PCR using the specific primer pair containing DNA recombination sequences, forward: 5’-GGGGACAAGTTTGTACAAAAAAGCAGGCTCCAACATGGCTGCCAAAGTGTTTGAGTCC-3’, and reverse: 5’-GGGGACCACTTTGTACAAGAAAGCTGGGTCCTACTGGGGCAGCTGGAGGAGCACG-3’. The PCR product was subcloned into an entry vector pDONR221 to generate pDONR221/PHB1, which was then recombined with a destination vector pLenti6.3/V5-DEST to create pLenti/PHB1. A commercially available plasmid pLenti/GFP, sharing the same backbone (Invitrogen), was used as a control. The mixture of pLenti plasmid and three packaging plasmids was cotransfected in 293FT cells by using ViraPower HiPerform Lentiviral Expression Systems (Invitrogen). The recombinant lentiviruses, Lenti/PHB1 and Lenti/GFP, in the culture medium were harvested, respectively. The harvested lentivirus was further concentrated using Lenti-X Concentrator (Clontech), and then titrated by determining the viral RNA genome content with Lenti-X qRT-PCR Titration kit (Clontech) according to the manufacture’s manual.

ASC were seeded in 35mm dishes at 1×10^5^cells/dish and allowed to recover overnight in ASC growth medium. The following day, the cell density was about 60% confluence. The medium was replaced with transduction medium, 300μl of growth medium containing lentivirus (MOI=2) and 8μg/ml polybrene (Sigma) per dish. After one hour incubation with rocking the dish back and forth every 20 minutes, 1.7ml of growth medium containing 8μg/ml polybrene was added. Following an overnight incubation, the medium was changed to fresh ASC growth medium. After additional incubation for two days, the over-confluent cells were treated with adipogenic medium (day 0) as described above. Transduction efficiency was evaluated by determining GFP expression of Lenti/GFP in ASC with flow cytometry. Because of the high transduction efficiency (always in excess of 80%), no antibiotic selection was required.

**Isolation of nuclei**

Extraction of nuclear lysate of 3T3-L1 cells was performed by using NE-PER nuclear and cytoplasmic extraction reagents (Thermo Scientific) based on our previous descriptions and the manufacturer’s instructions. The immunoblotting was performed as described in the main ‘Materials and Methods’. The primary antibody, anti-TATA binding protein (TBP), was bought from Abcam.

**References**

1. Gregory-Bass RC, Olatinwo M, Xu W, Matthews R, Stiles JK, et al. (2008) Prohibitin silencing reverses stabilization of mitochondrial integrity and chemoresistance in ovarian cancer cells by increasing their sensitivity to apoptosis. Int J Cancer 122: 1923-1930.

2. Liu D, Hou J, Hu X, Wang X, Xiao Y, et al. (2006) Neuronal chemorepellent Slit2 inhibits vascular smooth muscle cell migration by suppressing small GTPase Rac1 activation. Circ Res 98: 480-489.
